# Ladies First: Gender Stereotypes Drive Anticipatory Eye-Movements During Incremental Sentence Interpretation

**DOI:** 10.3389/fpsyg.2021.589429

**Published:** 2021-06-23

**Authors:** Ernesto Guerra, Jasmin Bernotat, Héctor Carvacho, Gerd Bohner

**Affiliations:** ^1^Center for Advanced Research in Education, Institute of Education, Universidad de Chile, Santiago, Chile; ^2^Center for Cognitive Interaction Technology, CITEC, Bielefeld University, Bielefeld, Germany; ^3^Department of Psychology, Bielefeld University, Bielefeld, Germany; ^4^School of Psychology, Pontificia Universidad Católica de Chile, Santiago, Chile

**Keywords:** gender stereotypes, language comprehension, anticipatory eye movements, explicit beliefs, eye tracking

## Abstract

Immediate contextual information and world knowledge allow comprehenders to anticipate incoming language in real time. The cognitive mechanisms that underlie such behavior are, however, still only partially understood. We examined the novel idea that gender attitudes may influence how people make predictions during sentence processing. To this end, we conducted an eye-tracking experiment where participants listened to passive-voice sentences expressing gender-stereotypical actions (e.g., “The wood is being painted by the florist”) while observing displays containing both female and male characters representing gender-stereotypical professions (e.g., florists, soldiers). In addition, we assessed participants’ explicit gender-related attitudes to explore whether they might predict potential effects of gender-stereotypical information on anticipatory eye movements. The observed gaze pattern reflected that participants used gendered information to predict who was agent of the action. These effects were larger for female- vs. male-stereotypical contextual information but were not related to participants’ gender-related attitudes. Our results showed that predictive language processing can be moderated by gender stereotypes, and that anticipation is stronger for female (vs. male) depicted characters. Further research should test the direct relation between gender-stereotypical sentence processing and implicit gender attitudes. These findings contribute to both social psychology and psycholinguistics research, as they extend our understanding of stereotype processing in multimodal contexts and regarding the role of attitudes (on top of world knowledge) in language prediction.

## Introduction

Humans frequently generate expectations about what will happen in the near future (e.g., thinking of tonight’s dinner) or even in the immediate subsequent moment. When processing language, listeners are often capable to predict the word upcoming in the next seconds or even milliseconds. Indeed, anticipatory processing (or prediction) in language and cognition has received special attention in recent years, partly as a consequence of extensive experimental evidence showing that listeners and readers can make online language predictions (see DeLong et al., 2014; Huettig, 2015 for reviews), but also triggered by a heated debate around mounting inconsistent evidence on the pervasiveness of this phenomenon (DeLong et al., 2017; Nieuwland et al., 2018; Huettig and Guerra, 2019; Nicenboim et al., 2020). Thus, while some level of prediction appears to occur, it is much less clear which cognitive mechanisms support this behavior and what is its role for language and cognition (see Friston, 2010; Hauk, 2016).

Experimental studies on language-mediated visual attention have shown that during spoken sentence comprehension, prediction can be triggered by unfolding linguistic cues and knowledge about the world, allowing comprehenders to anticipate to-be-mentioned visual referents in real time (see Knoeferle and Crocker, 2007; Borovsky et al., 2012). Interestingly, other studies have shown that listeners can rapidly make inferences based on speakers’ voices and related social stereotypes, such that comprehension is more difficult when subsequent language does not match the activated stereotype (van Berkum et al., 2008). Research suggests that information about people is frequently processed using stereotypes about social groups. For instance, people might attribute certain characteristics as intrinsic to a particular gender, e.g., women have lower mathematics abilities than men (Spencer et al., 1999; Dovidio et al., 2005; Jost and Kay, 2005).

Indeed, stereotypes about gender do affect sentence comprehension. Carreiras et al. (1996) asked participants to read sentence pairs such as “The footballer wanted to play in the match. He had been training very hard during the week.” In their study, the gender pronoun was manipulated to match (e.g., He) vs. mismatch (e.g., She) the gender stereotype associated with the role name (e.g., footballer), which resulted in faster vs. slower reading times, respectively (see also Duffy and Keir, 2004; Kreiner et al., 2008). These findings have been interpreted as suggesting that gender-stereotypical information triggers inferences based on people’s world knowledge (e.g., Kreiner et al., 2008). However, research in social psychology suggests otherwise: Although stereotypes constitute basic cognitive structures for categorization, they are in fact culturally transmitted and thus they reflect social biases rather than plausibility (see Brown, 2011).

Previous research on language prediction has certainly shown the relevance of real-world plausibility knowledge. In a study by Kamide et al. (2003), participants saw a visual display with two characters (e.g., tailor, plumber) and four objects (e.g., sewing machine, fabric, sink, and pipe). Participants’ eye movements were recorded as they inspected the displays and listened to recorded materials. Spoken sentences such as “The tailor will cut the fabric” would allow precise prediction of the ensuing object only if participants were able to use their world knowledge (about tailors) in addition to the lexical information provided by the verb (cf. Altmann and Kamide, 1999). Results showed that participants anticipated *fabric* instead of *pipe* when hearing “The tailor will cut the….” In turn, they preferred to look at the *pipe* upon hearing “The plumber will repair the….”

Thus, comprehenders can combine long-term memory representations (world knowledge) with incoming lexical (e.g., the verb) and visual (e.g., referents) inputs in real time to make predictions about what will be mentioned next (see also Knoeferle and Crocker, 2007; van Berkum et al., 2008; Borovsky et al., 2012). With sentences like those from Kamide et al. (2003), the top-down influence of world knowledge on verbal and visual information processing provides the comprehenders with sufficient constraints to unequivocally anticipate the correct object, both when the tailor cuts the fabric and when the plumber repairs the pipe. In such a case, certainly, comprehenders retrieve world knowledge about how occupations are associated with particular actions.

Research on the role of gender in language, however, has been somewhat different. First, although previous studies (e.g., Carreiras et al., 1996; Duffy and Keir, 2004; Kreiner et al., 2008) have looked into how gender-associated professions are integrated with a preceding linguistic context, they have not yet addressed the question of whether stereotypes are relevant for making predictions. This is the first question we address in the present study. Secondly, most studies have not directly contrasted stereotypes about women with stereotypes about men but have rather reported the overall processing costs of encountering counter-stereotypical information (cf., Cacciari and Padovani, 2007; Siyanova-Chanturia et al., 2012). It is, thus, unclear whether there are any specific biases for female- or male-stereotypical occupations and how they are integrated into preceding linguistic (and non-linguistic) context, another issue we will examine in our experiment.

### Gender Stereotypes Beyond World Knowledge

In the language comprehension literature, the use of gender stereotypes has often been treated as part of world knowledge (e.g., Kreiner et al., 2008). In social psychology, by contrast, studies suggest that people derive their attitudes and stereotypes *from* world knowledge (see Locksley et al., 1980). For instance, Koenig and Eagly (2014) showed that gender stereotypes can be built based on the observation of gender roles: Characteristics of roles that are occupied predominantly by women are attributed to women (e.g., nurses tend to be women, thus women are good at caring for people). A previous study (Hoffman and Hurst, 1990) showed that stereotypes emerge as rationalizations of existing role-related distributions even in the presence of extensive individuating information that is uncorrelated with either the roles or the stereotyped groups. Even more, after stereotypes are established, they can be impervious to real-world information and plausibility.

In a more recent study, Cao and Banaji (2016) tested whether gender-stereotypical associations could be overridden when factual information was provided. In three experiments, participants were presented with two names (e.g., Jonathan, Elizabeth) and were told that one of them was a doctor and the other a nurse. Participants’ beliefs about these two characters were assessed using an explicit and an implicit measure both before and after individuating facts were provided. Beliefs at the explicit level were evaluated by asking participants who the doctor was and who the nurse. At the implicit level, participants’ beliefs were measured using an Implicit Association Test (IAT, Greenwald et al., 1998), which measured the strength of association between each individual—Jonathan vs. Elizabeth—and the attribute of doctor vs. nurse. In all three experiments, gender stereotypes operated at the explicit and implicit levels before participants knew individuating facts; when asked about the characters’ professions, participants strongly preferred Jonathan to be the doctor and Elizabeth the nurse. Similarly, response times in the IAT matched the beliefs that Jonathan was in fact the doctor and Elizabeth the nurse. More importantly, after individuating factors were presented to the participants, their explicit beliefs were updated and participants responded in accordance with this new information, whereas their implicit beliefs continued to reflect the gender bias.

This leads to the question of whether the use of gendered information could be based on sexist attitudes and not just world knowledge. If only world knowledge drives the processing of gendered information in language, then predictions based on this information should be balanced for both genders. Thus, stereotypes about women should have the same status as stereotypes about men. However, if stereotypes about women are based on sexist attitudes (that is, giving women a lower status than men in society, see Fiske, 1993; Jost and Banaji, 1994), then gender-stereotypical language processing should work differently for stereotypes about women than for stereotypes about men.

### The Current Study

We ask whether listeners make use of gender stereotypes (both visually and verbally derived) to make predictions about the agent (that is, the doer or initiator of an action expressed by a verb; see Kroeger, 2005) of verbally conveyed actions. Moreover, if listeners do so, are female and male gender stereotypes treated in the same way? Furthermore, is gender-driven anticipation behavior related to individual differences in explicit beliefs about gender? These questions appear to be particularly important, considering that accounts of prediction during sentence processing have not integrated people’s attitudes, but have treated any informational biases as part of real-world knowledge.

To address these questions, we constructed male- and female-stereotypical visual and spoken materials (i.e., occupations and actions, respectively) and combined them to generate six experimental conditions. In each trial, participants saw a display with a male and a female character who, depending on the experimental condition, represented a male-stereotypical occupation (e.g., soldier) or a female-stereotypical occupation (e.g., florist). These were combined with one of three types of sentence in German: sentences conveying female-stereotypical actions (e.g., “The wood is being painted by the florist_female_,” “*Das Holz wird angemalt von der Floristin*”), or conveying male-stereotypical actions (e.g., “The wood is being cut by the florist_female_,” “*Das Holz wird gehackt von der Floristin*”), or a neutral sentence (e.g., “The wood is being stored by the florist_female_,” “*Das Holz wird gelagert von der Floristin*”), which served as a control condition. We predicted that if gender stereotypes guide anticipatory eye movements, we would observe preferential looks toward the stereotypical character before it is referred to. Moreover, we predicted that the effect of the linguistic stereotype (i.e., actions) on the anticipatory eye-movements would be moderated by the visual stereotype, namely the occupation that the characters in the visual display represent.

Finally, finding a relation between anticipatory eye movements and participants’ individual scores on explicit attitudes about gender would provide further support for the idea that the use of gender-stereotypical information during predictive language processing is based on sexist attitudes.

## Materials and Methods

### Participants

Fifty-one German native speakers (16 men; *M*_age_ = 23.57, age range: 18–36 years) from the University community, with normal or corrected-to-normal vision, participated. All participants gave informed consent and were paid either 2€ or given course credits and chocolates for their participation. The number of participants was based on sample sizes previously reported in the literature on predictive eye movements during spoken sentence comprehension (see, e.g., Altmann and Kamide, 1999; Kamide et al., 2003; Borovsky et al., 2012; Huettig and Guerra, 2019).

### Experimental Materials

**FIGURE 1:**
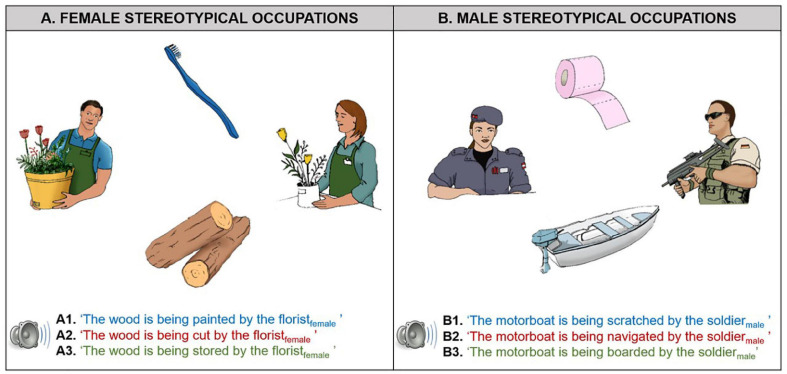
Visual context example as a function of visually derived gender stereotypes (**A**, female-stereotypical occupations; **B**, male-stereotypical occupations), as well as the corresponding verbally conveyed gender stereotypes (**1**. female-stereotypical actions in blue font; **2**. male-stereotypical occupations in red font; **3**. neutral in green font). On each trial, participants saw a visual context with either female- or male-stereotypical occupations and heard one out of three possible types of sentences. The objects (e.g., wood, motorboat) and the characters (e.g., florist_female_, soldier_male_) were always kept the same within an experimental item, while the sentential verb determined the gender stereotypical action.

A pre-test on gender associations of different occupations and actions was carried out to generate the verbal and visual materials. Norming data are presented in Supplementary Material. Forty-two unique displays were constructed to serve as the visual context in the eye-tracking experiment. Each of them presented a female and a male character representing the same occupation (e.g., a female and a male florist) and two objects, e.g., toothbrush and wooden logs (see Figure 1). A total of 14 occupations were used in the experiments, half of which were stereotypically associated with women (e.g., hairdresser, flight attendant), and half stereotypically associated with men (e.g., firefighter, guard).

For each of the 14 occupations, three passive sentences in German were constructed to match the displays, resulting in a total of 42 trials. The sentences always described an action that one of the characters acted upon one of the objects. The action was stereotypically associated with men (e.g., “The wood is being cut by the florist_female_,” see Figure 1A1), women (e.g., “The wood is being painted by the florist_female_” see Figure 1A2), or neutral (e.g., “The wood is being stored by the florist_female_” see Figure 1A3), depending on the critical verb. As can be seen in these examples, the agent (e.g., the florist_female_), or critical noun, was kept the same within each visual context. To balance the stimuli, participants listened to the same number of trials with female and male agents across items.

Additionally, 28 filler trials were also constructed, each with a unique visual context (also with two characters and two objects) and a passive sentence (e.g., “The cell phone is being charged by the firefighter,” *“Das Handy wird aufgeladen von dem Feuerwehrmann”*). Characters and objects were repeated among fillers, but none of the verbs from the experimental items were used. After each filler trial, a *yes*-or-*no* question about the sentences or the pictures was presented (e.g., “Is the tablet being charged by the fireman?” *“Wird das Tablet aufgeladen von dem Feuerwehrmann?”*), which served as a check on participants’ engagement on the task (see Supplementary Material for a full list of experimental items and filler materials).

### Experimental Design

Our experimental design combined two levels of visually derived gender-stereotypical occupations (i.e., female-stereotypical vs. male-stereotypical occupations) and three levels of verbally conveyed gender-stereotypical actions (i.e., female-stereotypical vs. male-stereotypical vs. neutral actions). We implemented a 2 × 3 repeated-measures Latin square design, which crossed all six experimental conditions in six experimental lists. Each participant saw one list with every visual context and heard a sentence in one experimental condition. Thus, every participant saw 42 experimental trials, with seven trials per condition. Finally, the same 28 filler trials were also presented on each experimental list. Trial presentation was pseudo-randomized for each participant. In that way, the first trial was always a filler and no more than two experimental items were presented consecutively.

### Attitudinal Measures

To assess participants’ explicit attitudes about gender, we used two standardized scales; the Normative Gender Role Orientation scales (NGRO, Athenstaedt, 2000) and the Ambivalent Sexism Inventory (ASI, Glick and Fiske, 1996; German-language version by Eckes and Six-Materna, 1999). The NGRO consists of 29 statements expressing participants’ attitudes toward normative gender roles in society (e.g., “Women are less interested in politics than men,” “*Frauen sind weniger an Politik interessiert als Männer*”). Participants indicated their agreement with each item on a scale from 1 = *does not apply* to 7 = *applies*). The ASI consists of two 11-item sub-scales, benevolent sexism (ASI-BS; e.g., “No matter how accomplished he might be, a man is not truly complete as a person without the love of a woman,” “*Egal, wie erfolgreich ein Mann auch sein mag, ohne eine Frau, die ihn liebt, fehlt ihm etwas ganz Wichtiges*”) and hostile sexism (ASI-HS; e.g., “Women are too easily offended,” *“Frauen sind zu schnell beleidigt”*). Participants indicated their agreement with each item on a scale from 0 (*I totally disagree*) to 5 (*I totally agree*). The internal consistencies for NGRO (Cronbach-α = 0.89), ASI-BS (Cronbach-α = 0.90), and ASI-HS (Cronbach-α = 0.89) in our sample were high.

### Procedure

After giving informed consent, participants first completed the ASI and then the NGRO scale. This took about 10 min. Then, the eye-tracking experiment began, which also took about 10 min to complete. Participants sat comfortably at approximately 70 cm from the computer screen and rested their chins on the eye tracker’s head support. Their eye movements were recorded using an Eyelink 1000 Plus Desktop Mount (SR Research) as they inspected a visual display and listened to linguistic materials through standard computer speakers on each trial. They were instructed to pay attention to what they were hearing and to what they were seeing, which is sometimes called “look-and-listen” studies (see Huettig et al., 2011). They also learned that, occasionally, a *yes*-or-*no* question about the sentences or the pictures would have to be answered. Before the beginning of the experiment, a default calibration procedure was carried out. On every trial, a participant began fixating a cue in the center of the screen, allowing the experimenter to initiate the trial (or re-calibrate whenever necessary). The visual display was presented for 3 s before the spoken sentences were presented.

## Data Analysis and Results

### Accuracy

Accuracy of responses to the engagement-check items was computed by assigning a zero to incorrect responses and a one to correct responses, and then calculating the mean for each participant. This yields the percentage of correct responses per participant. Participants’ accuracy on comprehension questions was at ceiling for most participants (*M* = 91%; range 79–100%), showing that they engaged in the experiment.

### Eye-Tracking Data

#### Data Analysis

To examine participants’ gaze behavior, four areas of interest (AOI) corresponding to the four displayed pictures were defined. Next, a trial-based summary of fixations was produced (Data Viewer software, SR research). This fixation report provided the duration and location of all eye fixations on each trial, which allowed the individualization of the fixation falling into the different interest areas. Subsequently, we used the R Project software (R Core Team, 2020) to further divide our data into time steps of 100 ms. To achieve that, we first inspected all fixations per participant and trial in time steps of 1 ms, where a value of 1 was given to the interest area fixated by the participant at each time step, and a value of 0 to all other areas. Afterward, we aggregated these short time windows by averaging 100 ms again at the participant, trial, experimental condition, and AOI levels. Finally, the average proportion at the participant level and the corresponding 95% confidence intervals (adjusted for within-subjects designs; see Morey, 2008) were calculated for each interest area on each experimental condition for each 100-ms time step. The average onset of the critical verb was 1719.26 ms (*SD* = 228.75 ms) before the onset of the critical noun. Thus, the extent of the time window of analysis goes from 2,000 ms before the onset of the critical noun to 1,000 ms after the onset of the critical noun. This 3,000 ms time window allowed us to determine whether participants exhibited any agent preference before the onset of the critical verb, between the onset of the critical verb and the onset of the critical noun (expected predictive effect), and after the onset of the critical noun (expected referential effect).

Inferential analysis was conducted through non-parametric cluster analysis based on random permutations of conditions (see Barr et al., 2014; Kronmüller et al., 2017; Kronmüller and Noveck, 2019). To do so, we first calculated the log-ratio (see Arai et al., 2007) between the proportion of fixation toward the female and male agents on each condition per participant per trial. Thus, positive log-transformed values represent a preference for the female agent in the visual context, whereas negative values reflect a preference for the male agent in the visual context, independently of experimental condition.

Cluster-based randomization analysis was performed in two stages. We first identified the clusters of interest, defined as a large epoch composed by consecutive 100 ms time windows with reliable effects. We assessed the log-ratio difference between the agents (i.e., female vs. male), as well as the difference between the log-ratio and a chance or zero distribution (i.e., a vector of zeros reflecting no object preference; see Barzy et al., 2020) for each gender-stereotypical action condition (i.e., female, male, and neutral) independently. Statistical significance (*p* < 0.05) for each 100-ms time window was assessed through mixed-effect linear regressions on our dependent variable (i.e., log-ratio) with stereotypical action (i.e., female vs. male) as fixed effect and random intercepts for participants and items, for each time window and visually derived gender-stereotypical occupation condition separately.

The second stage involved creating three null-hypothesis distributions of *t*-values, achieved by randomly permutating the values or labels that distinguish different levels of a factor (e.g., female- and male-stereotypical actions). Permutations are based on 2000 iterations in which every 100 ms time window is tested with the labels scrambled in the simulated experiments. Thus, no relation between experimental condition and data remains, providing the null-hypothesis *t* distributions. The first null-hypothesis distribution was generated by randomly rearranging the visual condition labels (i.e., female- and male-stereotypical occupations), which allowed us to compare the preference for the female agent against the preference for the male agent on each verbal condition individually. To generate the other two null-hypothesis distributions, we first created a chance (or zero) distribution given that log-ratio around zero expressed no object preference (see Barzy et al., 2020). We then created the second and the third null distributions by randomly permutating the female label in the visual condition with the zero label (from a chance distribution) and by permutating the male label in the visual condition with the zero label, respectively. Once the *t* distributions were computed, we aggregated the *t*-values at the cluster and iteration levels and then identified the largest absolute summed *t*-value per iteration and summed them for each cluster level. Finally, we assessed the statistical significance of clusters by comparing the sum of largest *t*-values of each empirically obtained cluster with the corresponding distribution of largest *t*-values generated in the simulation. A cluster was considered significant by two-tailed test if it was below percentile 2.5 in that distribution (see Chan et al., 2018).

#### Results

**FIGURE 2:**
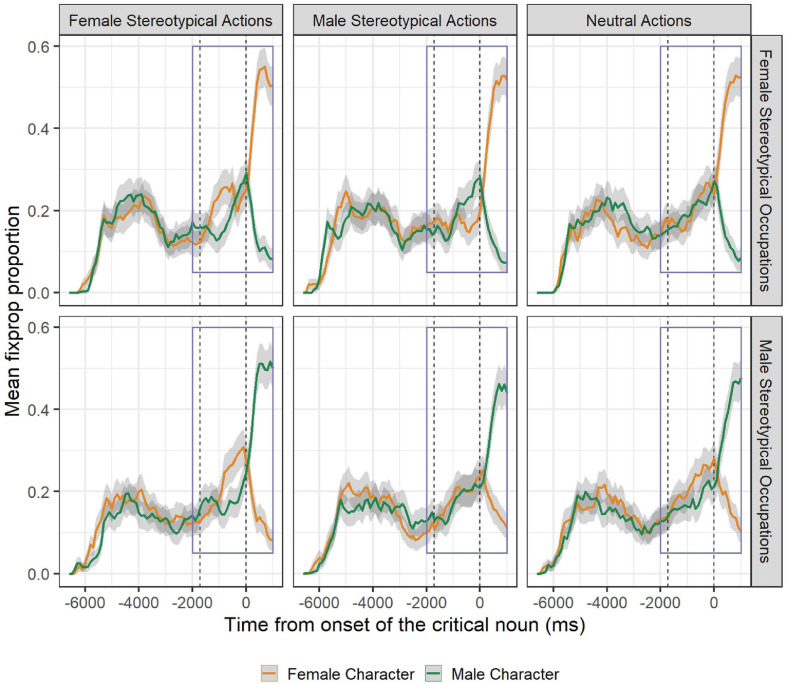
Mean proportion of fixation in all experimental conditions. In all panels, orange lines represent proportion of fixations toward the female character, and green lines represent fixations toward the male character. Gray areas around the main lines represent corresponding 95% CIs adjusted for within-subject designs. The first vertical dashed line represents average onset of the critical verb, and the second line marks the exact onset of the critical noun. The purple squares within the figures mark the window of analysis.

Figure 2 presents the time-course graphs with the mean proportion of fixations and corresponding adjusted confidence intervals for the critical time window in all six conditions of the 2 × 3 design. The proportions of fixations (and corresponding CIs) show that neutral sentences afforded no prediction and participants preferred one of the two characters only after the onset of the critical noun. In turn, when participants heard a sentence that described an action stereotypically associated with women, they preferred to look at the female character *before* the agent of that action was mentioned. These predictive patterns, however, differed depending on the visually derived gender-stereotypical occupations: Participants preferred the female agent 1,200 ms before the onset of the critical noun when they inspected a display with two characters representing an occupation stereotypically associated with women, but only 800 ms before the onset of the critical noun when they saw a display with two characters representing an occupation stereotypically associated with men.

When participants were presented with a visual context with female-stereotypical occupations and heard male-stereotypical action sentences, we again observed that participants anticipated a character based on the stereotypical action (i.e., the male character). However, the effect appeared only 500 ms before the onset of the critical noun. Finally, when participants were presented with a visual context with male-stereotypical occupations and heard male-stereotypical action sentences, no preferential looks were observed before the onset of the critical noun, resembling the neutral categories.

The results from the cluster-based randomization analysis are consistent with what can be directly inferred from the fixation-proportion time-course plots. In trials where participants heard a neutral sentence, significant clusters appeared only 200 ms after the onset of the critical noun (female agent preference: Observed sum *t* = 88.11; male agent preference: Observed sum *t* = 62.24, contrast between occupation conditions: Observed sum *t* = 129.93, all *p* < 0.001), independently of whether the agents represented an occupation typically associated with women or men.

By contrast, when participants heard a sentence conveying an action stereotypically associated with women, they exhibited a clear preference for the female agent in the visual context. This preference was significant from 1,200 to 600 ms (observed sum *t* = 17.7, *p* < 0.001) before the onset of the critical noun when participants were presented with a female-stereotypical occupation in the visual context. When they saw a male-stereotypical occupation in the visual context, their preference was somewhat delayed, beginning 800 ms and lasting until 100 ms before the onset of the critical noun (observed sum *t* = 22.21, *p* < 0.001). The differences on how female-stereotypical actions operated when visually situated in a male- vs. female-stereotypical occupation visual context was confirmed by two significant clusters that identified differences between agents’ preference in the two distinctive visual contexts (from −1300 to −1000 ms, observed sum *t* = 9.58, *p* < 0.01, and from −400 to −100 ms, observed sum *t* = 12.43, *p* < 0.001). After the onset of the critical noun, participants exhibited a clear preference for the mentioned agent (All clusters started at 200 ms after the noun onset; female agent preference: Observed sum *t* = 98.11; male agent preference: Observed sum *t* = 90.14, contrast between occupation conditions: Observed sum *t* = 161.66, all *p* < 0.001).

**FIGURE 3:**
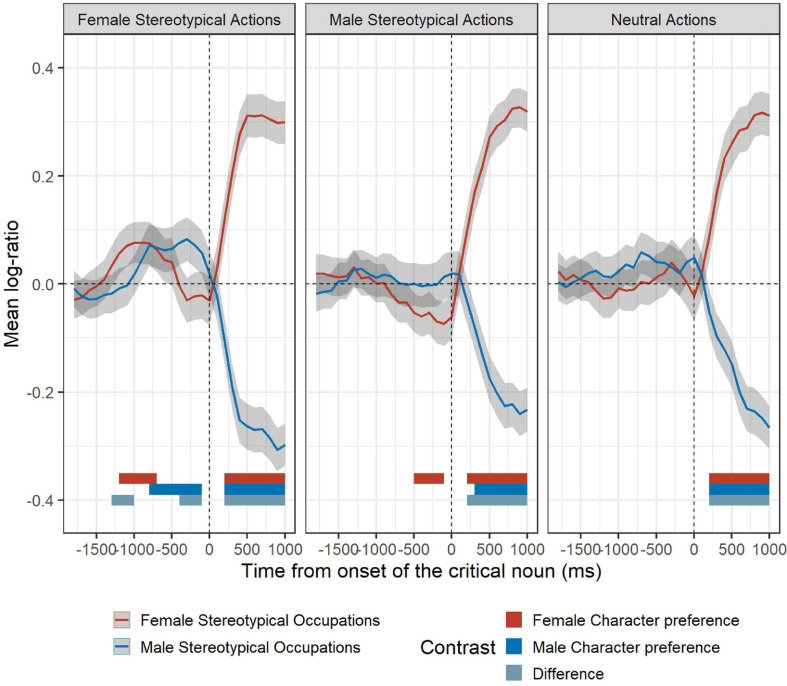
Mean log-ratio between the female and male agents (positive and negative values reflect preference for the female and male character, respectively), as a function of the action experimental condition (neutral actions, female-stereotypical actions, male-stereotypical actions), and aggregated in time steps of 100 ms. Plots are time-locked to the onset of the critical noun. Occupation experimental conditions (female-stereotypical occupations, male-stereotypical occupations) are represented by different line colors. Shaded areas represent within-subject adjusted 95% confidence levels, calculated at the participant level. Bottom horizontal bars visually represent the extent (in milliseconds) of identified significant cluster for each contrast and action experimental condition.

When participants heard a sentence expressing an action stereotypically associated with men, they attempted to anticipate the agent of the action (i.e., exhibited more looks to the male rather than the female character before any of them is mentioned), only if the visual context depicted two characters representing occupations typically associated with women. A significant cluster reflecting preference for the male agent was identified between 500 and 100 ms before the onset of the critical noun (observed sum *t* = 15.59, *p* < 0.001). This suggests that male-stereotypical action sentences have a different effect on anticipatory eye movements when situated in a male- vs. female-stereotypical occupation visual context. After the onset of the critical noun, participants preferred to look at the agent mentioned from 200 ms (observed sum *t* = 96.04, *p* < 0.001) in the visual female-stereotypical occupation context and from 300 ms in the visual male-stereotypical occupation context (observed sum *t* = 62.75, *p* < 0.001). The difference between visual contexts also emerged at 200 ms after critical noun onset (observed sum *t* = 135.59, *p* < 0.001). Figure 3 offers a visual depiction of the cluster analysis on log-ratio between female and male agents in each experimental condition. In particular, the horizontal bars at the bottom show the extension in time of clusters identified as significant for each experimental condition.

### Attitudes and Anticipatory Eye Movements

#### Data Analysis

Participants’ scores on the NGRO and the two ASI scales were *z*-standardized and used as predictors in an ordinary least squares (OLS) regression for the dependent variable derived from eye movements (i.e., mean log-ratio) aggregated per participant and cluster of interest (i.e., significant clusters occurring before the onset of the critical noun). As we had identified three clusters of interest—that is, a window of time during which we see significant differences between conditions—the condition to which each cluster pertained (i.e., female-stereotypical action in a female-stereotypical occupation visual context, female-stereotypical action in a male-stereotypical occupation visual context, and male-stereotypical action in a female-stereotypical occupation visual context) was also introduced in the regression model as a non-ordinal three-level fixed effect.

As can be seen in Figure 3, the male-stereotypical action in a female-stereotypical occupation condition produced anticipation toward the male character, and thus the mean log-ratio values tended to be negative (whereas the female-stereotypical action condition triggered looks to the female character and thus mean log-ratio values tended to be positive). Therefore, participants’ mean log-ratio values on that cluster were multiplied by −1 for a more informative comparison between clusters. Our OLS regression uses the data from each scale (by participant), experimental condition, and their interaction to jointly predict the mean log-ratio for each cluster of interest. We conducted three equivalent models (identical predictors), each with a different cluster as reference group, by changing only the contrast coding for the cluster predictor levels.

#### Results

Contrary to our expectations, we observed no main effects of the attitude measures on anticipatory eye movements on any of the clusters, no differences between clusters, and no interaction effects (all *t*-values < | 2|). Table 1 presents a subset of the results from the OLS regression analysis. Concretely, we present the estimates and corresponding statistics for the effect of each scale on each cluster, which we achieved conducting the regression model with different contrast codings, setting the intercept to each cluster. The distributions of the scales and clusters, as well as their intercorrelations, are presented in Supplementary Material.

**TABLE 1:** Ordinary least square regression results. Within the table (in bold font), the experimental condition of the cluster that served as intercept in each model.

	**Estimate**	***Se***	***t***	***Pr(*> *|t|)***
**Female-stereotypical action and occupation**				
(Intercept)	0.070	0.021	3.405	<0.001***
NGRO	−0.033	0.029	−1.139	0.257
ASI (BS)	−0.005	0.025	−0.209	0.834
ASI (HS)	0.008	0.031	0.259	0.796
**Male-stereotypical action and female-stereotypical occupation**				
(Intercept)	0.062	0.021	3.009	0.003**
NGRO	0.029	0.029	1.003	0.318
ASI (BS)	−0.008	0.025	−0.316	0.752
ASI (HS)	0.003	0.031	0.089	0.929
**Male-stereotypical action and occupation**				
(Intercept)	0.069	0.021	3.378	<0.001***
NGRO	0.000	0.029	0.009	0.993
ASI (BS)	−0.040	0.025	−1.582	0.116
ASI (HS)	−0.007	0.031	−0.215	0.830

***p < 0.001; **p < 0.01.

## Discussion

Substantial experimental evidence has shown that gender stereotypes do indeed moderate processing time during sentence understanding (Carreiras et al., 1996; Duffy and Keir, 2004; Kreiner et al., 2008). However, we identified a couple of open issues in the context of gender stereotypes. First, no previous experiments had investigated whether gender stereotypes are relevant for predictive language processing as reflected in anticipatory eye movements. Second, we noticed that in the literature on language comprehension, gender-stereotype effects have been interpreted as comprehenders’ use of their world knowledge. Literature in social psychology, however, suggests that stereotypes and world knowledge are not equivalent (see, e.g., Cao and Banaji, 2016). Instead, whereas world knowledge is routinely used by people to derive (gender) stereotypes and attitudes, factual counter-stereotypical information might be integrated only at an explicit level but not an implicit level, despite plausibility.

We addressed these questions using a well-established eye-tracking paradigm (Altmann and Kamide, 1999), which allowed us to identify whether participants would anticipate a visual referent based on stereotypical information and to dissociate the moment-by-moment effects of gender stereotypes about women and men during language comprehension. The results showed that participants used gender stereotypes in language and the scene in real time to predict the agent of the sentence. Interestingly, this anticipation was not symmetrical for female and male stereotypes. Analysis of the gaze patterns’ time course during language comprehension revealed earlier and longer predictive eye movements when sentences conveyed female stereotypes than when sentences conveyed male stereotypes. Moreover, verbally conveyed gender stereotypes (i.e., actions) interacted with the visually derived stereotypes (i.e., occupations represented by the characters). Anticipatory eye-movements to the female character occurred 300 ms earlier when female-stereotypical action sentences were accompanied by female-stereotypical occupations (e.g., florist) than when those sentences were presented together with male-stereotypical occupations (e.g., soldier). Similarly, when male-stereotypical action sentences were presented together with female-stereotypical occupations we observed relatively late anticipatory eye-movements toward the male character, already 300 ms later than when participants heard female-stereotypical action sentences and were looking at male-stereotypical occupations.

These findings are consistent with a view in which sexism moderates predictive sentence processing. Gender stereotypes rapidly triggered anticipatory eye movements, in particular when visually and linguistically derived representations were consistent, and less so when they were in conflict. Moreover, these effects appear to have had a distinct time course for female- and male-stereotypical information. Although not predicted, we conjecture that this asymmetry in our findings is consistent with a view of women (as opposed to men) being the main targets of sexism. Indeed, negative stereotypes of women (see Glick and Fiske, 1996) are more pervasive and central for male-dominated societies (see e.g., Sidanius and Pratto, 2001; but cf. Eagly and Mladinic, 1994). Consequently, if sexism drives anticipatory eye-movements, it might be responsible for the earlier and larger effect for female-stereotypical action sentences relative to male-stereotypical action sentences that we observed.

The pattern observed for male-stereotypical action sentences when presented together with male-stereotypical occupations seems, at first glance, more intriguing. Although the visual depictions and sentences were stereotypically consistent (both male-biased), no predictive effect was observed at all. We argue, however, that this finding as well may be seen as consistent with the notion that sexist bias can drive prediction during language processing. It is also consistent with literature on gender stereotypes outside of the language-processing domain: When processing gendered information, participants are biased to routinely check whether such information is aligned with female stereotypes (see Glick and Fiske, 1996). From this viewpoint, in our task the listener did not evaluate who was the character that was more likely to be the agent of the action described. Rather, the listener may have assessed (in real time) how likely it is that the female character would be performing the action described. Consequently, when the male-stereotypical action sentences were presented together with the female character impersonating a male-stereotypical occupation (and thus, not conforming with the gender stereotype), she was not less likely to perform a male-stereotypical action relative to the male character, and thus we observed no anticipatory eye movements.

This mechanism is also consistent with the advantage observed for the female-stereotypical action sentences; a female-stereotypical occupation is perceived as being highly likely to perform a female-stereotypical action, whereas a counter-stereotypical female character is perceived as less so. Finally, this mechanism could also explain the delayed predictive effect found in the male-stereotypical action sentence with female-stereotypical occupation; as the sentence unfolded, listeners discarded the female character as the likely agent of the action, and only then they predicted the male-character to be more likely.

In this context, it would be reasonable to expect that the anticipatory preference for agents at the individual level should be related to individual differences in NGRO and ASI; however, we failed to find such effects (see Table 1). It is important to note that the NGRO and ASI scales assess explicit beliefs, that is, participants consciously reported their agreement with statements that are related to normative gender roles and sexist attitudes toward women. By contrast, anticipatory eye movements toward objects in a visual context during spoken language comprehension may be described as indicating an implicit attitude, at least to the extent that the representations activated by language automatically direct overt attention to related depicted objects. Thus, although explicit beliefs and attitudes about gender appeared to have no effect on predictive eye movements, we cannot rule out that such behavior would be related to gender bias as assessed by implicit measures of sexism (e.g., the IAT; see Glick and Fiske, 1996; Rudman et al., 2001). If such a correlation were found in future research, it would corroborate our interpretation. Also, given the unexpected nature of the asymmetry in predictive eye-movements caused by target gender, replication studies specifically addressing this aspect would be welcome.

In sum, we showed that predictive language processing is moderated by gender stereotypes. Importantly, we also found that this prediction is stronger for female (vs. male) depicted characters, consistent with a view in which sexism affects sentence processing incrementally. These findings contribute to both social psychology and psycholinguistics research, insofar as they extend our understanding of stereotype processing in multimodal contexts and with regard to the role of attitudes (on top of world knowledge) in language prediction.

## Data Availability Statement

All data and analysis scripts are available online in https://osf.io/asdfn.

## Ethics Statement

The studies involving human participants were reviewed and approved by Bielefeld University Ethics Committee. The patients/participants provided their written informed consent to participate in this study.

## Author Contributions

EG, JB, and GB developed the study concept. EG and JB implemented the experiment and analyzed the data. JB collected the data. EG and HC drafted the manuscript and it was revised by GB and JB. All authors interpreted the results and approved its final version for submission.

## Conflict of Interest

The authors declare that the research was conducted in the absence of any commercial or financial relationships that could be construed as a potential conflict of interest.
