# Supplementary material for: Ladies First: Gender Stereotypes Drive Anticipatory Eye-Movements During Incremental Sentence Interpretation
Source: Front Psychol. 2021 Jun 23;12:589429. doi: 10.3389/fpsyg.2021.589429 (PMC8279744; doi:10.3389/fpsyg.2021.589429)
Supplement: Supplementary file 1 [file Data_Sheet_1.docx]

**Supplementary Materials**

**Ladies first: Gender stereotypes drive anticipatory eye-movement during incremental sentence interpretation**

Ernesto Guerra^1,*^, Jasmin Bernotat^2,3^, Héctor Carvacho^4^, Gerd Bohner^3^

*^1^Center for Advanced Research in Education, Institute of Education, Universidad de Chile; ^2^Center for Cognitive Interaction Technology-CITEC, Bielefeld University; ^3^Department of Psychology, Bielefeld University; ^4^School of Psychology, Pontificia Universidad Católica de Chile*

** Corresponding author: [ernesto.guerra@ciae.uchile.cl](mailto:ernesto.guerra@ciae.uchile.cl)*

**Material norms**

We pre-tested a set of nouns referring to occupations and a set verb-object pairs, to assure that our materials clearly stand for each experimental condition. We constructed a list of occupations and verb-object pairs that were likely to be more strongly associated with either women or men (or not, for the neutral verb-object), from a stereotypically point of view, and ask 78 participants (53 women, 24 men, and one participant who did not indicate his/ her gender, age range: 19-38, *M*= 24.0, *SD*=4.03), to rate through a Likert 7-points Likert scale. To avoid order and magnitude effects, lists were fully randomized, and in half of the list the response scale was 1 for “typically male” and 7 for “typically female”, while on the other half the scale was inverted (1 for “typically female” and 7 for “typically male”). Participants were instructed to estimate (using the Likert scale) the extent to which society expects the presented actions and occupations to be performed by men or women. In Table S1, present the mean score for the 14 chosen occupations (out of 93 tested). Table S2 shows the mean score for all evaluated object-verb pairs. Table S3 and S4 presents the final 42 experimental sentences (based on our norming) and the 28 filler sentences (and corresponding yes-or-no questions), respectively.

**Table S1.** Mean score for the 14 selected occupation in the adjusted* Likert scale.

| Item | Occupation | | *M* |
| --- | --- | --- | --- |
| 1 | Bauarbeiter | Bauarbeiterin | 1.75 |
| 2 | Türsteherin | Türsteher | 1.92 |
| 3 | Soldat | Soldatin | 1.96 |
| 4 | Feuerwehrmann | Feuerwehrfrau | 2.00 |
| 5 | Mechatroniker | Mechatronikerin | 2.25 |
| 6 | Metzger | Metzgerin | 2.61 |
| 7 | Goldgräberin | Goldgräber | 2.65 |
|  |  |  |  |
| 8 | Florist | Floristin | 5.58 |
| 9 | Hausmann | Hausfrau | 5.58 |
| 10 | Flugbegleiterin | Flugbegleiter | 5.79 |
| 11 | Friseur | Friseurin | 5.79 |
| 12 | Zahnarzthelferin | Zahnarzthelfer | 5.83 |
| 13 | Parfümeriefachverkäuferin | Parfümeriefachverkäufer | 5.92 |
| 14 | Kosmetikerin | Kosmetiker | 6.35 |
| ** Since scale were inverted for half of the participants, we adjusted back the scale ((score+8)*-1) on that half. Thus, in the adjusted scale the larger the mean score, the more “typically female” the occupation.* | | | |

**Table S2.** Mean score for the 126 verb-object combinations in the adjusted* Likert scale.

|  |  |  | VERB | | |  |  |
| --- | --- | --- | --- | --- | --- | --- | --- |
| Item | OBJECT | Male | *M* | Neutral | *M* | Female | *M* |
| 1 | Auto | reparieren | 2.08 | anschauen | 2.57 | beschädigen | 4.38 |
| 2 | Backofen | warten | 3.46 | vorheizen | 5.35 | putzen | 5.46 |
| 3 | Bett | zusammenbauen | 2.79 | belegen | 4.83 | beziehen | 5.04 |
| 4 | Bier | trinken | 2.92 | verschütten | 3.21 | bringen | 4.61 |
| 5 | Cocktail | anbieten | 3.58 | belächeln | 3.75 | erwarten | 5.00 |
| 6 | Computer | programmieren | 2.08 | nutzen | 3.71 | zerstören | 3.92 |
| 7 | Fenster | einsetzen | 1.96 | schließen | 4.75 | schmücken | 5.83 |
| 8 | Fleisch | grillen | 2.21 | verzehren | 2.67 | zubereiten | 3.71 |
| 9 | Flugzeug | fliegen | 2.42 | bewundern | 2.87 | begleiten | 5.21 |
| 10 | Fußball | schießen | 2.54 | holen | 2.50 | verfehlen | 5.00 |
| 11 | Gabelstapler | beladen | 1.79 | beachten | 2.58 | übersehen | 4.21 |
| 12 | Geld | verdienen | 3.83 | finden | 3.83 | ausgeben | 4.59 |
| 13 | Grill | bedienen | 2.25 | kaufen | 2.42 | reinigen | 2.54 |
| 14 | Handtasche | missachten | 3.00 | öffnen | 6.00 | favorisieren | 6.08 |
| 15 | Holz | hacken | 1.79 | lagern | 2.08 | anmalen | 3.33 |
| 16 | Kartoffeln | ernten | 3.46 | essen | 3.79 | kochen | 4.83 |
| 17 | Kind | bespaßen | 5.08 | beschenken | 4.50 | bemuttern | 5.67 |
| 18 | Klavier | stimmen | 3.75 | spielen | 4.29 | wünschen | 4.71 |
| 19 | Kleid | komplimentieren | 5.33 | stehlen | 5.33 | anziehen | 6.50 |
| 20 | Kuscheltier | verbrennen | 3.58 | geben | 4.92 | umarmen | 5.35 |
| 21 | Lampe | anbringen | 2.54 | einschalten | 4.17 | polieren | 4.46 |
| 22 | Leuchtturm | orten | 2.92 | besuchen | 3.71 | bestaunen | 4.54 |
| 23 | Liebesroman | verbrennen | 4.52 | einsortieren | 5.75 | lesen | 6.17 |
| 24 | Maus | fangen | 3.00 | füttern | 4.61 | bekreischen | 5.58 |
| 25 | Motorboot | steuern | 2.50 | betreten | 3.04 | zerkratzen | 3.13 |
| 26 | Nagellack | ignorieren | 2.50 | umwerfen | 6.13 | empfehlen | 6.50 |
| 27 | Pfeife | rauchen | 1.78 | aufbewahren | 2.71 | dulden | 3.33 |
| 28 | Pferd | verarzten | 4.42 | striegeln | 4.96 | reiten | 5.25 |
| 29 | Pokerspiel | verfolgen | 2.21 | erklären | 2.38 | verachten | 4.83 |
| 30 | Raumschiff | erkunden | 2.38 | sichten | 2.83 | meiden | 4.05 |
| 31 | Rechnung | bezahlen | 3.54 | wahrnehmen | 3.83 | verursachen | 4.46 |
| 32 | Regal | montieren | 2.75 | auswählen | 4.96 | entstauben | 5.17 |
| 33 | Reifen | wechseln | 1.92 | bestellen | 2.08 | vergessen | 4.74 |
| 34 | Schraubenzieher | verwenden | 2.79 | wegräumen | 3.21 | anreichen | 3.67 |
| 35 | Schuhe | zerschleißen | 2.82 | tragen | 4.13 | anhimmeln | 6.13 |
| 36 | Sofa | bepolstern | 2.83 | aussuchen | 4.65 | dekorieren | 5.50 |
| 37 | Spinne | entfernen | 2.67 | sehen | 5.17 | fürchten | 5.29 |
| 38 | Stricknadeln | zweckentfremden | 3.91 | verschenken | 5.71 | benutzen | 5.79 |
| 39 | Teleskop | aufstellen | 2.96 | verwahren | 3.29 | umstoßen | 4.21 |
| 40 | Tisch | anfertigen | 2.29 | reservieren | 4.29 | decken | 5.13 |
| 41 | Waffe | abfeuern | 2.54 | entdecken | 2.79 | ablehnen | 4.75 |
| 42 | Wimperntusche | vermarkten | 6.21 | verwischen | 6.46 | auftragen | 6.63 |
| ** Since scale were inverted for half of the participants, we adjusted back the scale ((score+8)*-1) on that half. Thus, in the adjusted scale the larger the mean score, the more “typically female” the object-verb action.* | | | | | | | |

**Table S3.** Final experimental sentences based on our norming data.

|  |  |  | Main Action | | |  |
| --- | --- | --- | --- | --- | --- | --- |
| Item | Occupation | OBJECT + Aux. VERB | Female | Neutral | Male | AGENT |
| 1 | male | Das Auto wird | beschädigt | repariert | angeschaut | von dem Mechatroniker. |
| 2 | female | Der Backofen wird | geputzt | gewartet | vorgeheizt | von der Hausfrau. |
| 3 | female | Das Bett wird | bezogen | zusammengebaut | belegt | von der Flugbegleiterin. |
| 4 | male | Das Bier wird | gebracht | getrunken | verschüttet | von dem Türsteher. |
| 5 | female | Der Cocktail wird | erwartet | angeboten | belächelt | von der Flugbegleiterin. |
| 6 | female | Der Computer wird | zerstört | programmiert | genutzt | von der Parfümeriefachverkäuferin. |
| 7 | male | Das Fenster wird | geschmückt | eingesetzt | geschlossen | von dem Goldgräber. |
| 8 | male | Das Fleisch wird | zubereitet | gegrillt | verzehrt | von dem Metzger. |
| 9 | male | Das Flugzeug wird | begleitet | geflogen | bewundert | von dem Soldaten. |
| 10 | male | Der Fußball wird | verfehlt | geschossen | geholt | von dem Türsteher. |
| 11 | male | Der Gabelstapler wird | übersehen | beladen | beachtet | von dem Bauarbeiter. |
| 12 | female | Das Geld wird | ausgegeben | verdient | gefunden | von der Floristin. |
| 13 | male | Der Grill wird | gereinigt | bedient | gekauft | von dem Bauarbeiter. |
| 14 | male | Die Handtasche wird | favorisiert | missachtet | geöffnet | von dem Goldgräber. |
| 15 | female | Das Holz wird | angemalt | gehackt | gelagert | von der Floristin. |
| 16 | female | Die Kartoffeln werden | gekocht | geerntet | gegessen | von der Hausfrau. |
| 17 | female | Das Kind wird | bemuttert | beschenkt | bespaßt | von der Zahnarzthelferin. |
| 18 | female | Das Klavier wird | gewünscht | gestimmt | gespielt | von der Friseurin. |
| 19 | female | Das Kleid wird | angezogen | komplimentiert | gestohlen | von der Zahnarzthelferin. |
| 20 | female | Das Kuscheltier wird | umarmt | verbrannt | gegeben | von der Parfümeriefachverkäuferin. |
| 21 | male | Die Lampe wird | poliert | angebracht | eingeschaltet | von dem Mechatroniker. |
| 22 | male | Der Leuchtturm wird | bestaunt | geortet | besucht | von dem Feuerwehrmann. |
| 23 | male | Der Liebesroman wird | gelesen | verbrannt | einsortiert | von dem Türsteher. |
| 24 | male | Die Maus wird | bekreischt | gefangen | gefüttert | von dem Feuerwehrmann. |
| 25 | male | Das Motorboot wird | zerkratzt | gesteuert | betreten | von dem Soldaten. |
| 26 | female | Der Nagellack wird | empfohlen | ignoriert | umgeworfen | von der Kosmetikerin. |
| 27 | male | Die Pfeife wird | geduldet | geraucht | aufbewahrt | von dem Metzger. |
| 28 | male | Das Pferd wird | geritten | verarztet | gestriegelt | von dem Goldgräber. |
| 29 | female | Das Pokerspiel wird | verachtet | verfolgt | erklärt | von der Floristin. |
| 30 | female | Das Raumschiff wird | gemieden | erkundet | gesichert | von der Flugbegleiterin. |
| 31 | female | Die Rechnung wird | verursacht | bezahlt | wahrgenommen | von der Hausfrau. |
| 32 | female | Das Regal wird | entstaubt | montiert | ausgewählt | von der Friseurin. |
| 33 | male | Die Reifen werden | vergessen | gewechselt | bestellt | von dem Mechatroniker. |
| 34 | male | Der Schraubenzieher wird | angereicht | verwendet | weggeräumt | von dem Bauarbeiter. |
| 35 | female | Die Schuhe werden | angehimmelt | zerschlissen | getragen | von der Kosmetikerin. |
| 36 | female | Das Sofa wird | dekoriert | bepolstert | ausgesucht | von der Parfümeriefachverkäuferin. |
| 37 | female | Die Spinne wird | gefürchtet | entfernt | gesehen | von der Friseurin. |
| 38 | male | Die Stricknadeln werden | benutzt | zweckentfremdet | verschenkt | von dem Metzger. |
| 39 | male | Das Teleskop wird | umgestoßen | aufgestellt | verwahrt | von dem Feuerwehrmann. |
| 40 | female | Der Tisch wird | gedeckt | angefertigt | reserviert | von der Zahnarzthelferin. |
| 41 | male | Die Waffe wird | abgelehnt | abgefeuert | entdeckt | von dem Soldaten. |
| 42 | female | Die Wimperntusche wird | aufgetragen | vermarktet | verwischt | von der Kosmetikerin. |

**Table S4.** Filler sentences and corresponding yes-or-no engagement questions used in the experiment.

| Filler item and question | SENTENCE |  |
| --- | --- | --- |
| 1 | Das UFO wird gelandet von dem Floristen. |  |
|  | Wird das UFO geparkt von dem Floristen? |  |
| 2 | Die Kamera wird gemocht von der Hausfrau. |  |
|  | Trug der Hausmann eine Schürze? |  |
| 3 | Die Gitarre wird beklebt von dem Flugbegleiter. |  |
|  | Wird die Geige beklebt von dem Flugbegleiter? |  |
| 4 | Die Wäscheklammer wird befestigt von der Fri-seurin. |  |
|  | War die Wäscheklammer grün? |  |
| 5 | Die Teekanne wird gefüllt von dem Zahnarzthel-fer. |  |
|  | Wird die Tasse gefüllt von dem Zahnarzthelfer? |  |
| 6 | Der Hammer wird geschleudert von der Parfü-meriefachverkäuferin. |  |
|  | Hatte der Parfümeriefachverkäufer blonde Haare? |  |
| 7 | Die Zahnbürste wird angeschlossen von dem Kosmetiker. |  |
|  | Wird die Zahnbürste angeschlossen von dem Kosmetiker? |  |
| 8 | Der Vogel wird besungen von der Bauarbeiterin. |  |
|  | Hatte der Vogel schwarzes Gefieder? |  |
| 9 | Die Flasche wird genommen von dem Türsteher. |  |
|  | Wird die Flasche genommen von dem Floristen? |  |
| 10 | Die Hose wird gerissen von der Soldatin. |  |
|  | War auf dem Bildschirm eine Gitarre zu sehen? |  |
| 11 | Das Handy wird aufgeladen von dem Feuerwehr-mann. |  |
|  | Wird das Tablet aufgeladen von dem Feuerwehr-mann? |  |
| 12 | Der Topf wird erhitzt von der Mechatronikerin. |  |
|  | Hatte der Topf einen Deckel? |  |
| 13 | Die Tasse wird geleert von dem Metzger. |  |
|  | Wird die Tasse geleert von dem Metzger? |  |
| 14 | Der Stift wird graviert von der Goldgräberin. |  |
|  | War der Stift blau? |  |
| 15 | Die Leiter wird angesägt von der Floristin. |  |
|  | Wird der Baum angesägt von der Floristin? |  |
| 16 | Das Toilettenpapier wird aufgewickelt von dem Hausmann. |  |
|  | Hatte der Hausmann ein Bügelbrett? |  |
| 17 | Der Baum wird gepflanzt von der Flugbegleitern. |  |
|  | Wird der Baum gepflanzt von der Flugbegleite-rin? |  |
| 18 | Die Kopfhörer werden aufgesetzt von dem Fri-seur. |  |
|  | Waren die Kopfhörer schwarz? |  |
| 19 | Der Kinderwagen wird geschoben von der Zahn-arzthelferin. |  |
|  | Wird der Puppenwagen geschoben von der Zahn-arzthelferin? |  |
| 20 | Der Brunnen wird gespendet von dem Parfü-meriefachverkäufer. |  |
|  | Hatte der Parfümeriefachverkäufer eine Parfüm-flasche in der Hand? |  |
| 21 | Die Flöte wird versteckt von der Kosmetikerin. |  |
|  | Wird die Flöte versteckt von der Kosmetikerin? |  |
| 22 | Das Zelt wird bewohnt von dem Bauarbeiter. |  |
|  | War das Zelt rot? |  |
| 23 | Die Skier werden verstaut von der Türsteherin. |  |
|  | Werden die Schlüssel verstaut von der Türstehe-rin? |  |
| 24 | Der Rasenmäher wird geprüft von dem Soldaten. |  |
|  | War auf dem Bildschirm eine Toilettenpapierrolle zu sehen? |  |
| 25 | Die Harke wird aufgehoben von der Feuerwehr-frau. |  |
|  | Wird die Harke aufgehoben von der Feuerwehr-frau? |  |
| 26 | Der Vogel wird dirigiert von dem Mechatroniker. |  |
|  | Trug der Mechatroniker eine rote Jacke? |  |
| 27 | Die Flasche wird verschickt von der Metzgerin. |  |
|  | Wird die Post verschickt von der Metzgerin? |  |
| 28 | Die Kopfhörer werden reguliert von dem Gold-gräber. |  |
|  | Hatte der Goldgräber eine Schaufel in der Hand? |  |
| Note. Questions referred either to the previous picture or to the sentence. | | |


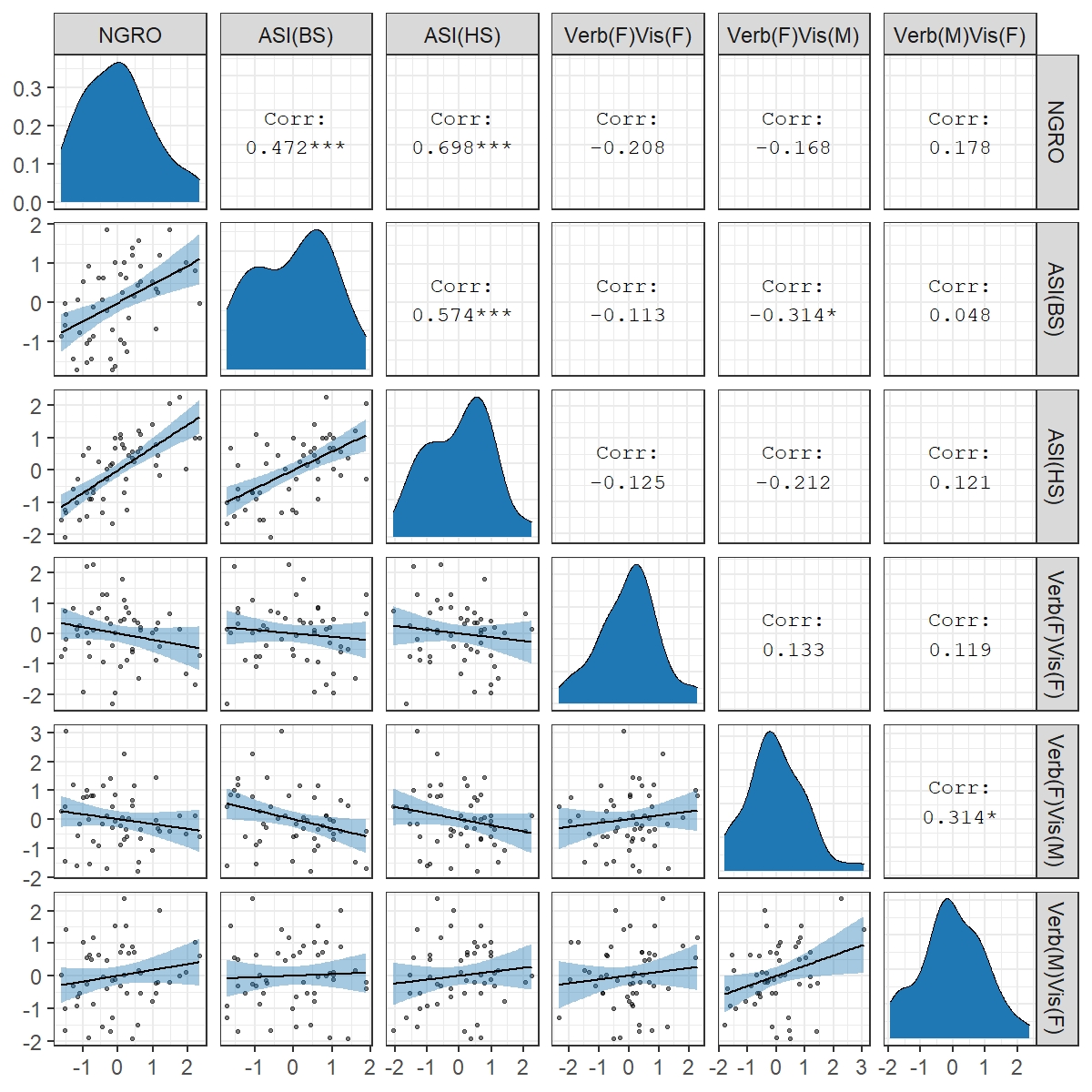


**Figure S1.** Distributions and the pairwise correlations for the NGRO and ASI scales (*** = *p* < .001; ** = *p <* .01; * = *p* < .05).
